# Supplementary material for: Simultaneous Degradation Study of Isomers in Human Plasma by HPLC-MS/MS and Application of LEDA Algorithm for Their Characterization
Source: Int J Mol Sci. 2022 Oct 28;23(21):13139. doi: 10.3390/ijms232113139 (PMC9653966; doi:10.3390/ijms232113139)
Supplement: Supplementary file 1 [file ijms-23-13139-s001.zip › ijms-1968757-supplementary.pdf]

Supplementary Material

Simultaneous Degradation Study of Isomers in Human Plasma by HPLC-MS/MS and Application of LEDA Algorithm for Their Characterization

Marco Pallecchi [1], Laura Braconi [1], Marta Menicatti [1], Sara Giachetti [2], Silvia Dei [1], Elisabetta Teodori [1] and Gianluca Bartolucci [1,*]

[1] NEUROFARBA Department, Section of Pharmaceutical and Nutraceutical Sciences, University of Florence, Polo Scientifico, Via U. Schiff 6, Sesto Fiorentino, 50019 Firenze, Italy

[2] Chemistry Department DICUS 'Ugo Schiff', University of Florence, Polo Scientifico, Via della Lastruccia 3, Sesto Fiorentino, 50019 Firenze, Italy

* Correspondence: gianluca.bartolucci@unifi.it

Table S1. The chromatographic parameters retention times (Rt) ± Error (2 standard deviation or 2 SD), base peaks width (Base Width), efficiency evaluated as the number of theoretical plates (N) and related relative standard deviation (RSD %) for each analyte obtained apply the proposed HPLC-MS/MS approach.

	Rt (min)	2 SD (min)	Base Width (min)	2 SD (min)	N	RSD (%)
IS	3.14	0.02	0.18	0.02	4804	10%
FRA76	3.35	0.02	0.19	0.02	4909	8%
GDE5	3.35	0.02	0.20	0.02	4472	7%
ELF94	3.53	0.02	0.19	0.02	5689	10%
ELF96	3.53	0.02	0.19	0.02	5511	9%

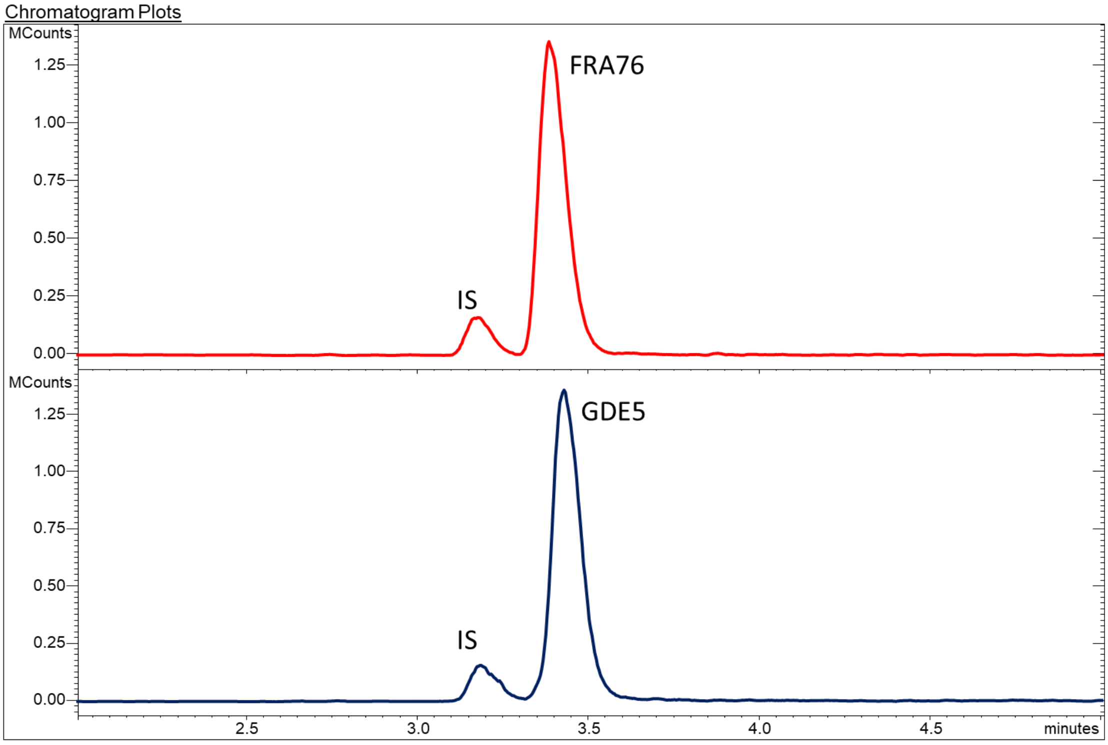

Figure S1. Chromatographic profiles of the HPLC-MS/MS analysis of the FRA76 and GDE5 isomers.

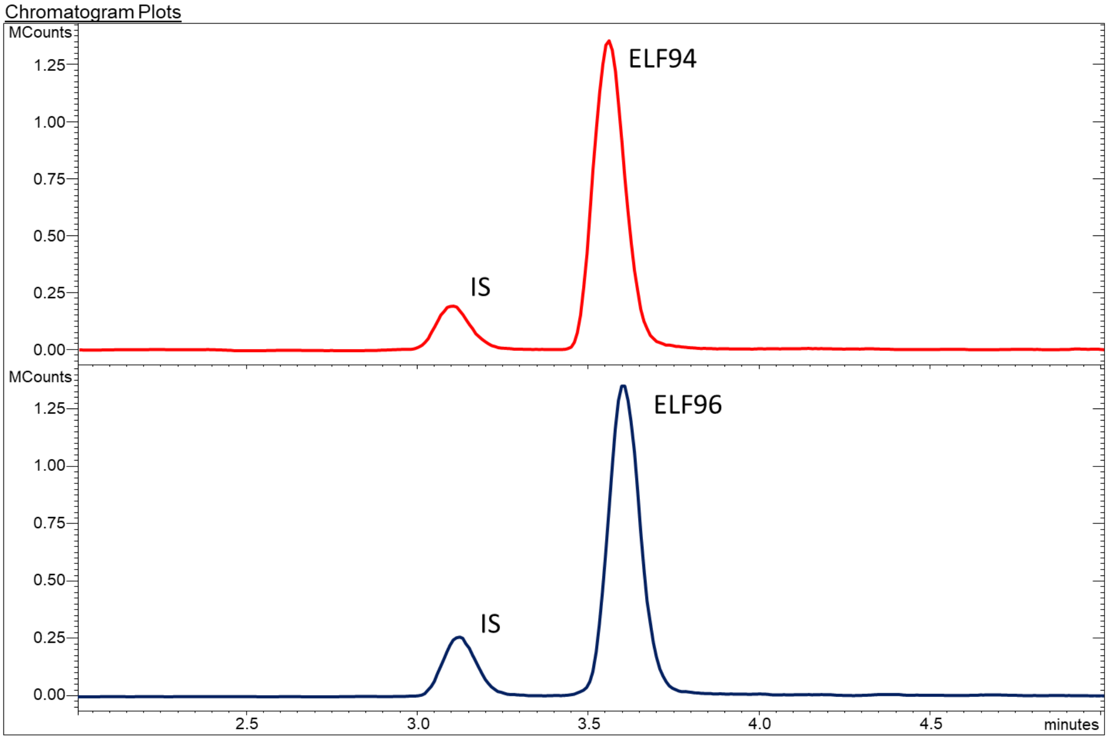

Figure S2. Chromatographic profiles of the HPLC-MS/MS analysis of the ELF94 and ELF96 isomers.

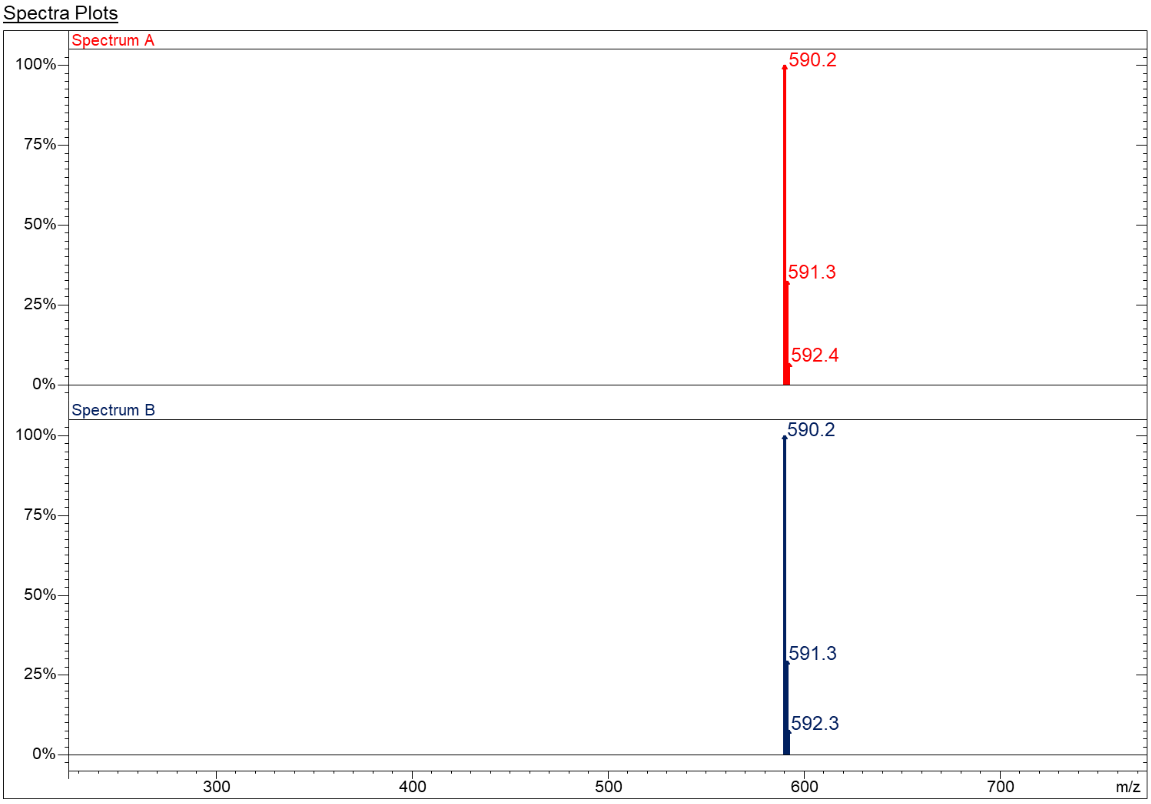

Figure S3. ESI-MS spectra of the FRA76 (spectrum A) and GGE5 (spectrum B) isomers.

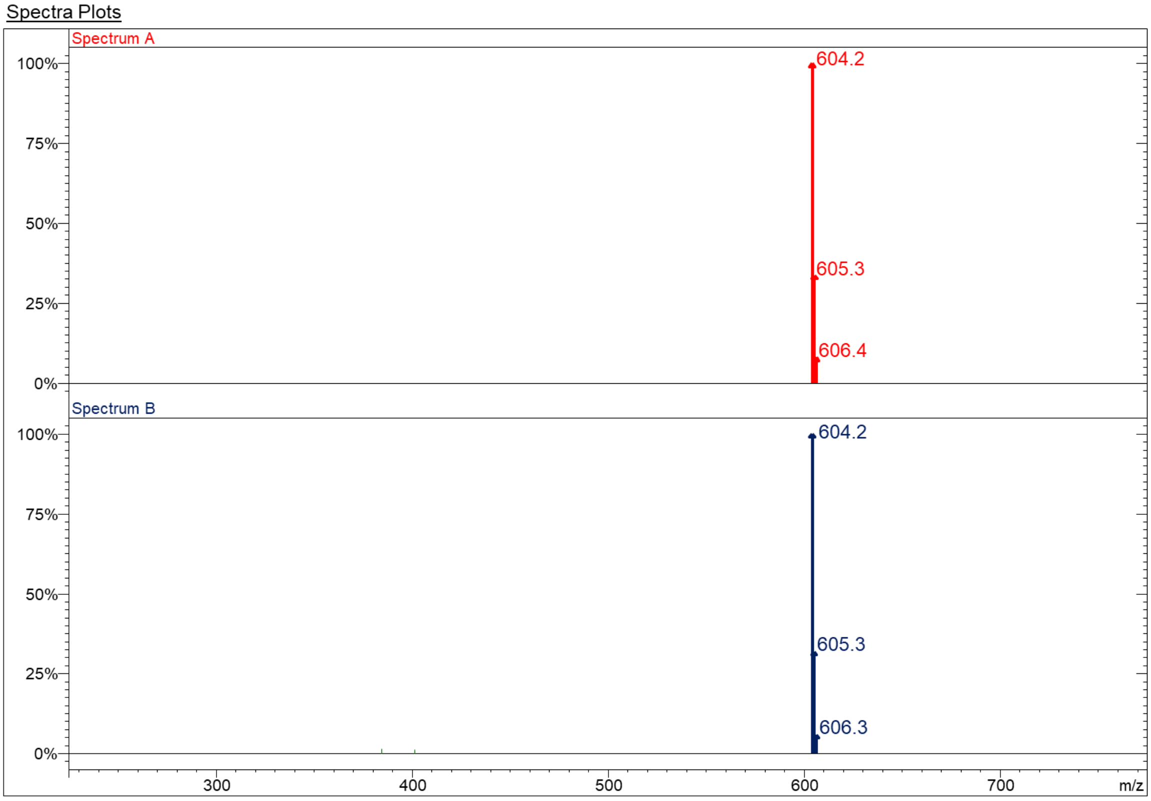

Figure S4. ESI-MS spectra of the ELF94 (spectrum A) and ELF96 (spectrum B) isomers.

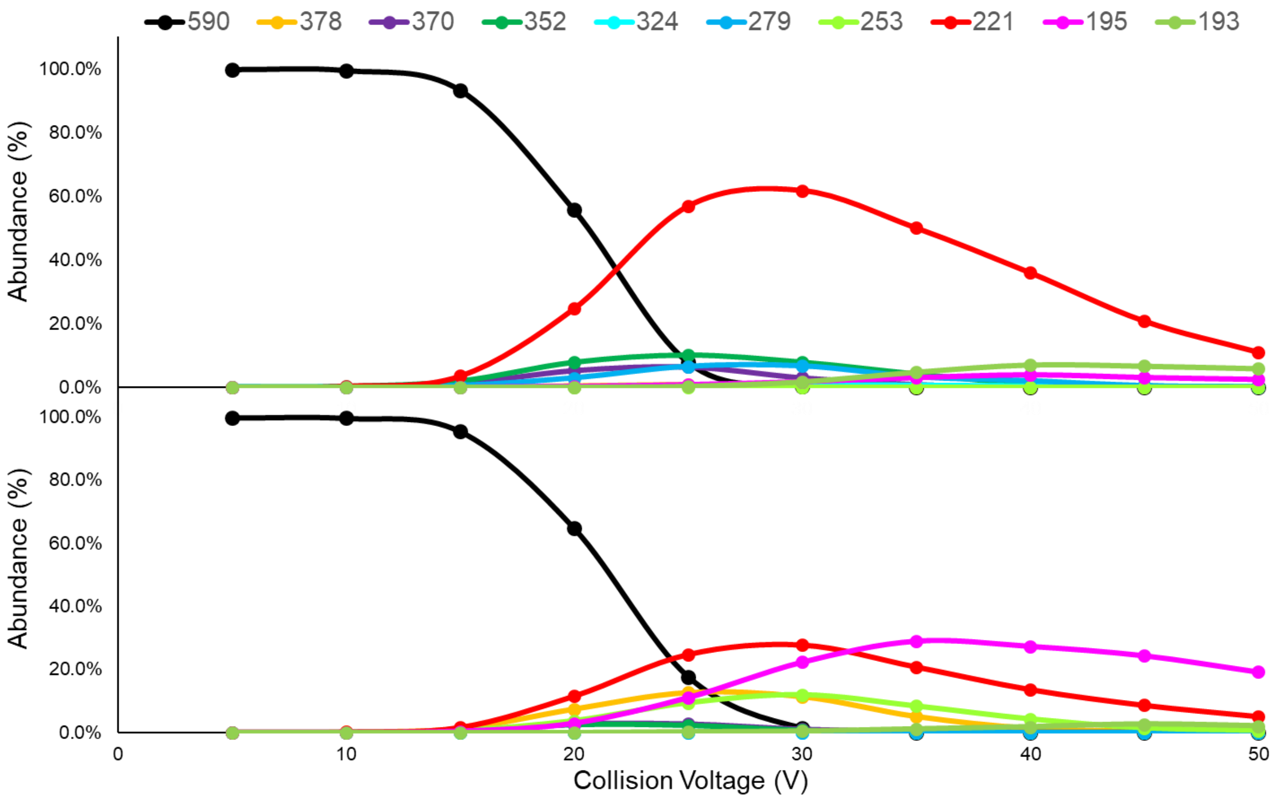

Figure S5. Comparison between the breakdown curves of selected MS/MS transitions from the FRA76 (top) and GDE5 (bottom) isomers.

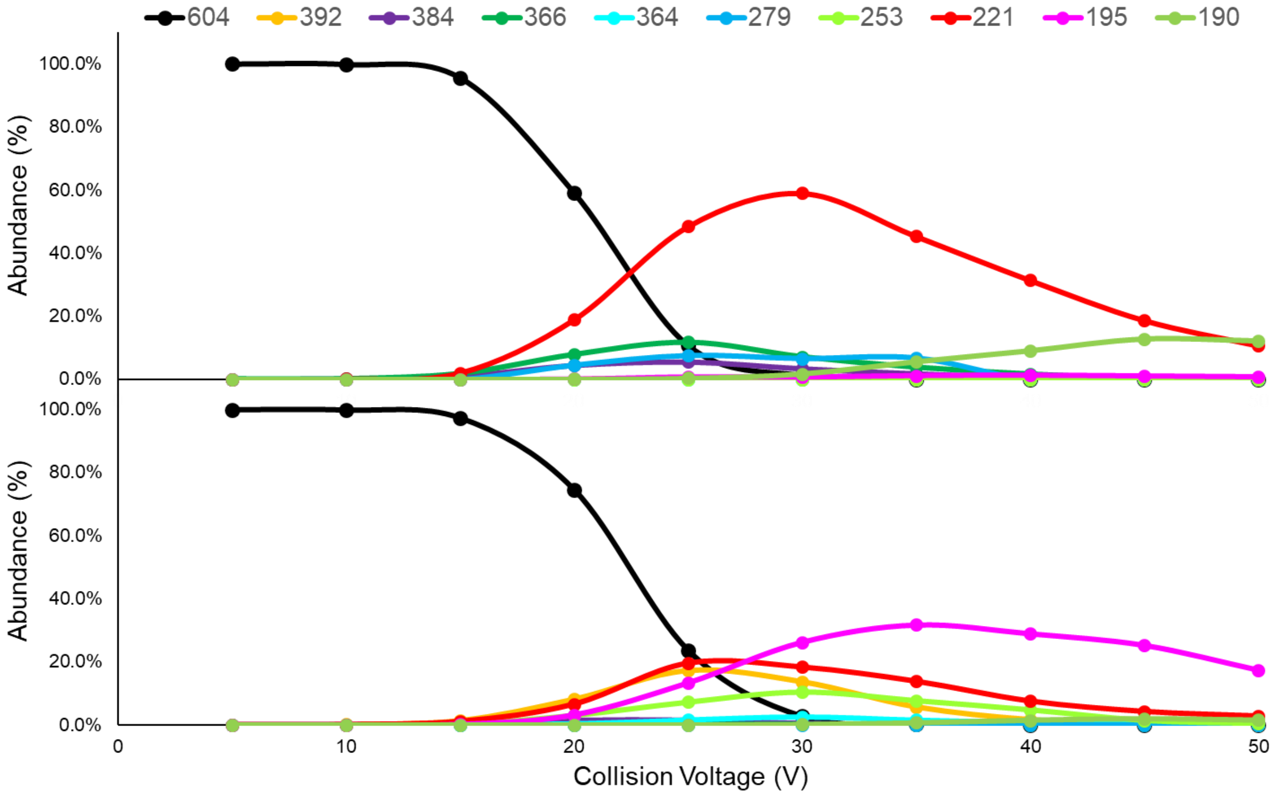

Figure S6. Comparison between the breakdown curves of selected MS/MS transitions from the ELF94 (top) and ELF96 (bottom) isomers.

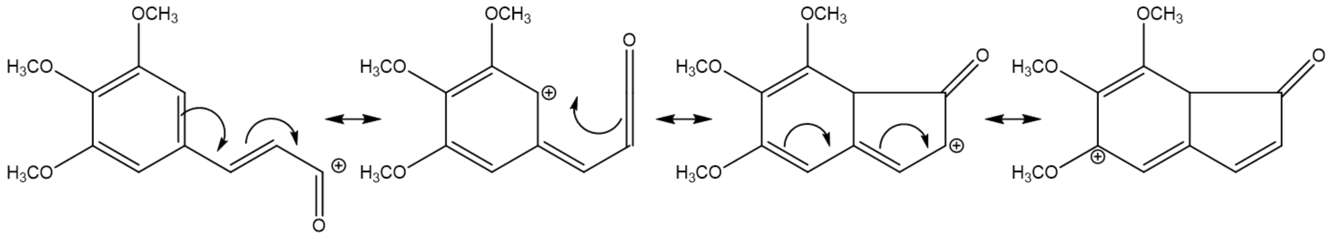

Chemical Formula: $C_{12}H_{13}O_4^+$
Exact Mass: 221.08

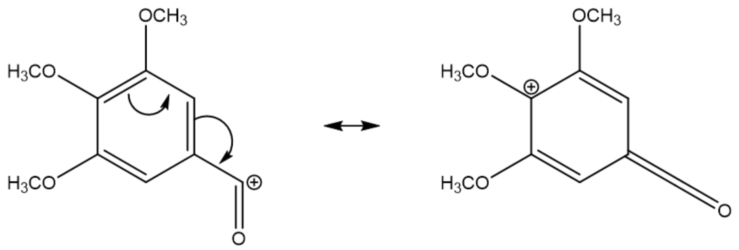

Chemical Formula: $C_{10}H_{11}O_4^+$
Exact Mass: 195.07

Figure S7. The proposed molecular structures of Pi_1 and Pi_2.

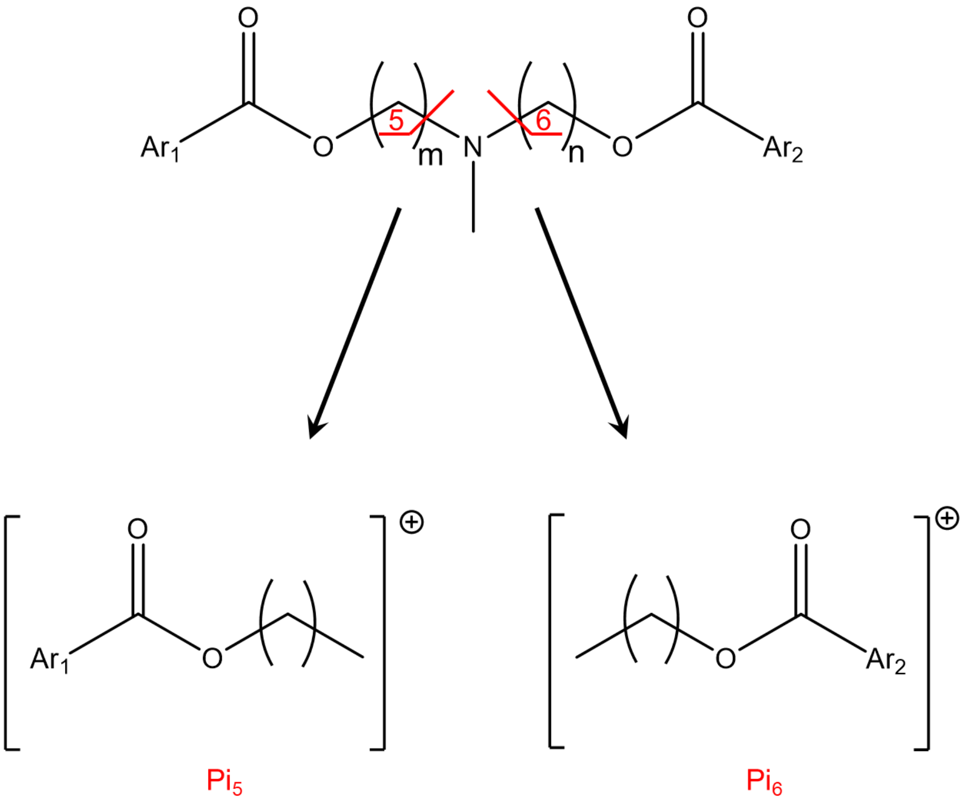

Figure S8. The fragmentation hypothesis of the studied P-gp inhibitors with formation of Pi_5 and Pi_6. Each bond cleavage site is marked by a red line and associated with a number, that is reported also to the related product ion (Pi).

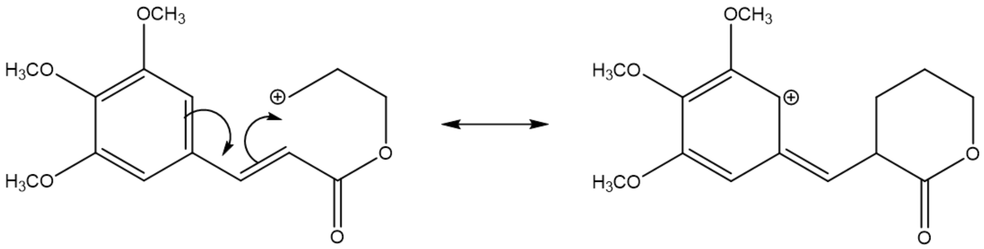

Chemical Formula: $C_{15}H_{19}O_5^+$
Exact Mass: 279.12

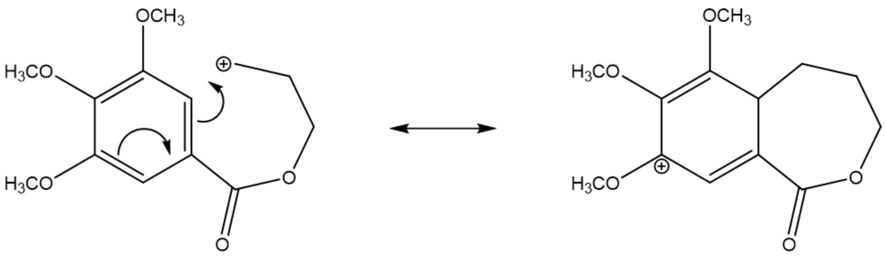

Chemical Formula: $C_{13}H_{17}O_5^+$
Exact Mass: 253.11

Figure S9. The propose molecular structures of Pi_5 and Pi_6.

S1 LEDA algorithm

LEDA195-221 square matrix for isomers speciation.

$$\begin{bmatrix} Area_{195}/Area_{Ri} = \left({Pi_2}/{Ri}\right)_{isomer\ 1}\ [\%]_{isomer\ 1} + \left({Pi_2}/{Ri}\right)_{isomer\ 2}\ [\%]_{isomer\ 2} \\ Area_{221}/Area_{Ri} = \left({Pi_1}/{Ri}\right)_{isomer\ 1}\ [\%]_{isomer\ 1} + \left({Pi_1}/{Ri}\right)_{isomer\ 2}\ [\%]_{isomer\ 2} \end{bmatrix} \text{(Equation S1)}$$

$$\begin{bmatrix} Area_{195}/Area_{Ri} \\ Area_{221}/Area_{Ri} \end{bmatrix} = A$$

$$\begin{bmatrix} \left({Pi_2}/{Ri}\right)_{isomer\ 1} & \left({Pi_2}/{Ri}\right)_{isomer\ 2} \\ \left({Pi_1}/{Ri}\right)_{isomer\ 1} & \left({Pi_1}/{Ri}\right)_{isomer\ 2} \end{bmatrix} = K$$

$$[[\%]_{isomer\ 1} \quad [\%]_{isomer\ 2}] = X$$

$$X = (A\,) \times (K\,)^{-1} \qquad \text{(Equation S2)}$$

LEDAAll matrix for isomers speciation.

$$\begin{bmatrix} Area_{195}/Area_{Ri} = \left({Pi_2}/{Ri}\right)_{isomer\ 1}\ [\%]_{isomer\ 1} + \left({Pi_2}/{Ri}\right)_{isomer\ 2}\ [\%]_{isomer\ 2} \\ Area_{221}/Area_{Ri} = \left({Pi_1}/{Ri}\right)_{isomer\ 1}\ [\%]_{isomer\ 1} + \left({Pi_1}/{Ri}\right)_{isomer\ 2}\ [\%]_{isomer\ 2} \\ Area_{253}/Area_{Ri} = \left({Pi_6}/{Ri}\right)_{isomer\ 1}\ [\%]_{isomer\ 1} + \left({Pi_6}/{Ri}\right)_{isomer\ 2}\ [\%]_{isomer\ 2} \\ Area_{279}/Area_{Ri} = \left({Pi_5}/{Ri}\right)_{isomer\ 1}\ [\%]_{isomer\ 1} + \left({Pi_5}/{Ri}\right)_{isomer\ 2}\ [\%]_{isomer\ 2} \\ Area_{Pi_3}/Area_{Ri} = \left({Pi_3}/{Ri}\right)_{isomer\ 1}\ [\%]_{isomer\ 1} + \left({Pi_3}/{Ri}\right)_{isomer\ 2}\ [\%]_{isomer\ 2} \\ Area_{Pi_4}/Area_{Ri} = \left({Pi_4}/{Ri}\right)_{isomer\ 1}\ [\%]_{isomer\ 1} + \left({Pi_4}/{Ri}\right)_{isomer\ 2}\ [\%]_{isomer\ 2} \end{bmatrix} \quad \text{(Equation S3)}$$

$$\begin{bmatrix} Area_{195}/Area_{Ri} \\ Area_{221}/Area_{Ri} \\ Area_{253}/Area_{Ri} \\ Area_{279}/Area_{Ri} \\ Area_{Pi_3}/Area_{Ri} \\ Area_{Pi_4}/Area_{Ri} \end{bmatrix} = A$$

$$\begin{bmatrix} \left({}^{Pi_2}/_{Ri}\right)_{isomer\ 1} & \left({}^{Pi_2}/_{Ri}\right)_{isomer\ 2} \\ \left({}^{Pi_1}/_{Ri}\right)_{isomer\ 1} & \left({}^{Pi_1}/_{Ri}\right)_{isomer\ 2} \\ \left({}^{Pi_6}/_{Ri}\right)_{isomer\ 1} & \left({}^{Pi_6}/_{Ri}\right)_{isomer\ 2} \\ \left({}^{Pi_5}/_{Ri}\right)_{isomer\ 1} & \left({}^{Pi_5}/_{Ri}\right)_{isomer\ 2} \\ \left({}^{Pi_3}/_{Ri}\right)_{isomer\ 1} & \left({}^{Pi_3}/_{Ri}\right)_{isomer\ 2} \\ \left({}^{Pi_4}/_{Ri}\right)_{isomer\ 1} & \left({}^{Pi_4}/_{Ri}\right)_{isomer\ 2} \end{bmatrix} = K$$

$$[[\%]_{Isomer\ 1} \quad [\%]_{isomer\ 2}] = X$$

Traspose K matrix = K'

Inverse $(K \times K')$ = $(K \times K')^{-1}$

$$X = (A \times K') \times (K \times K')^{-1} \qquad \text{(Equation S4)}$$

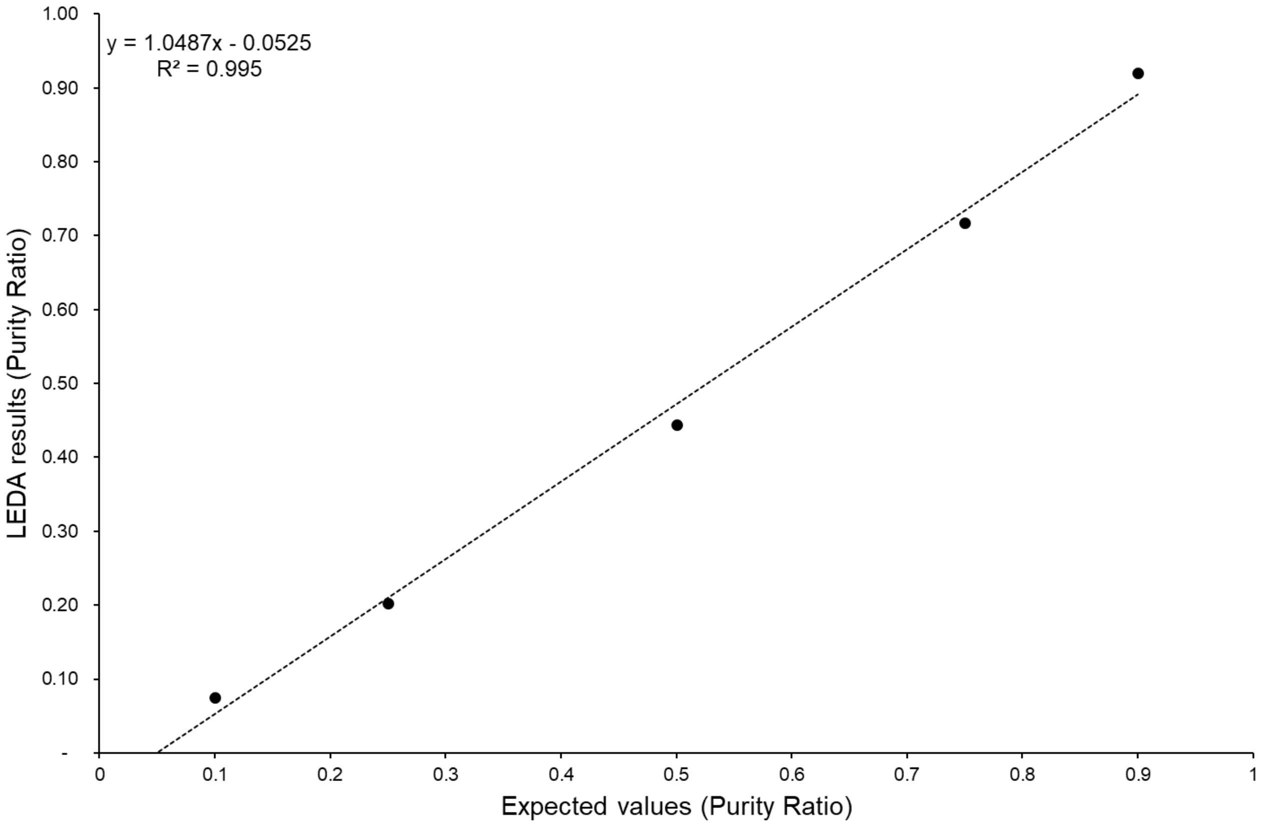

Figure S10. Validation plot obtained for the FRA76 isomer by processing the standard mixtures with LEDA$_{All}$ matrix.

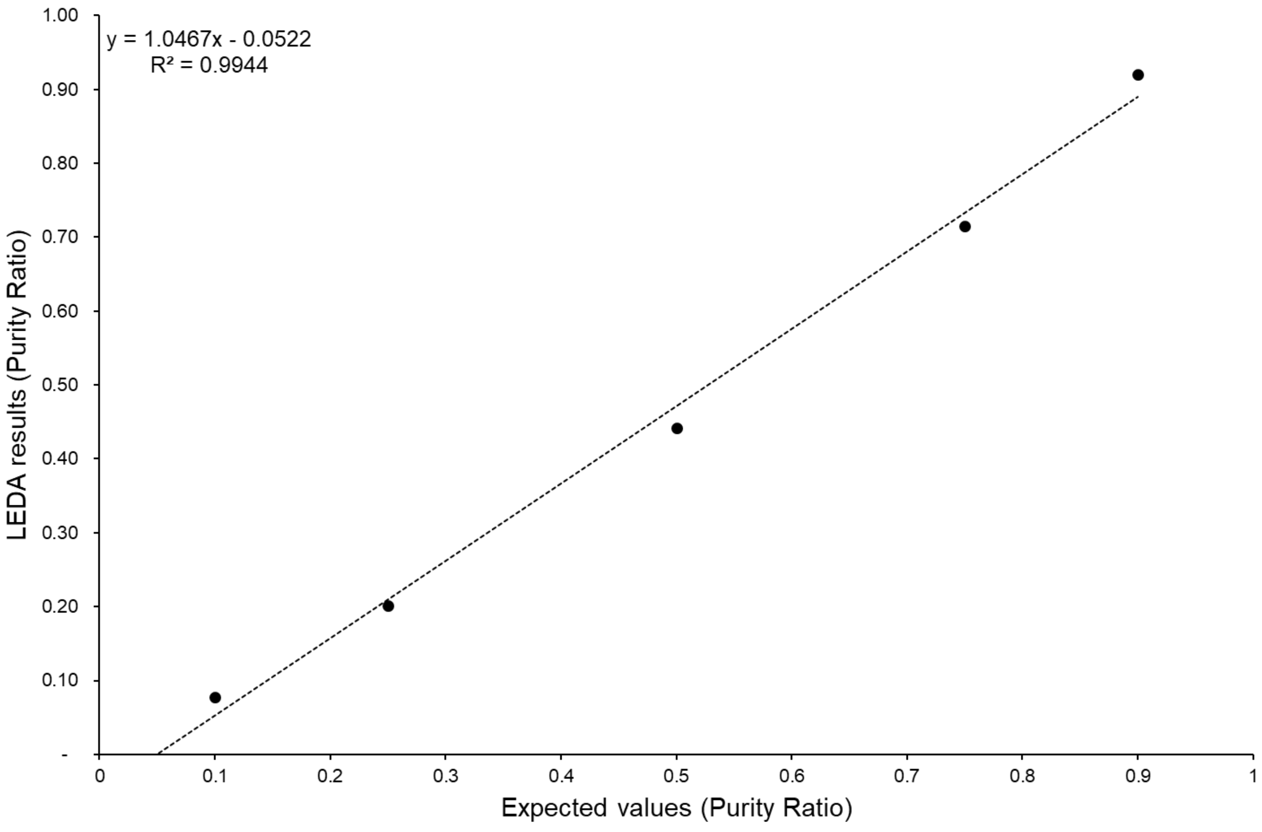

Figure S11. Validation plot obtained for the FRA76 isomer by processing the standard mixtures with LEDA$_{195\text{-}221}$ matrix.

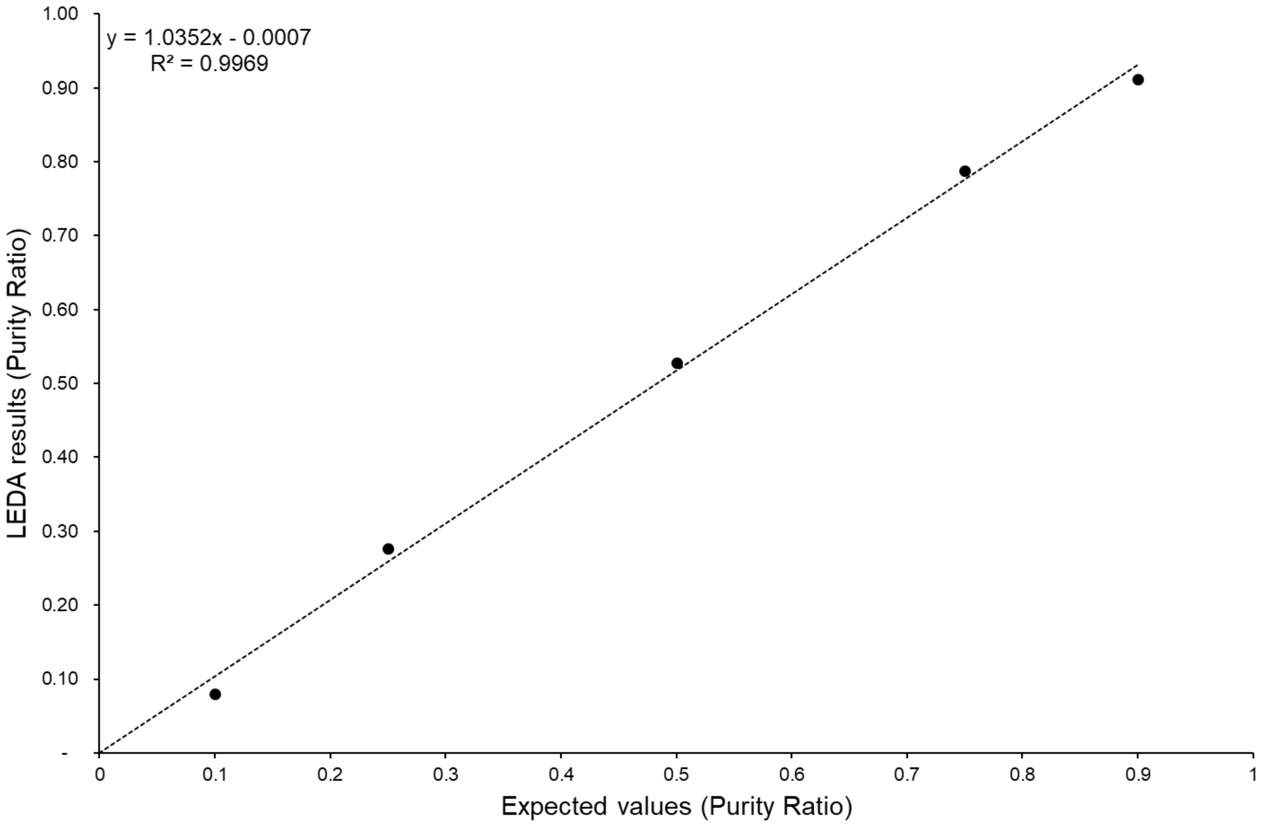

Figure S12. Validation plot obtained for the GDE5 isomer by processing the standard mixtures with $LEDA_{All}$ matrix.

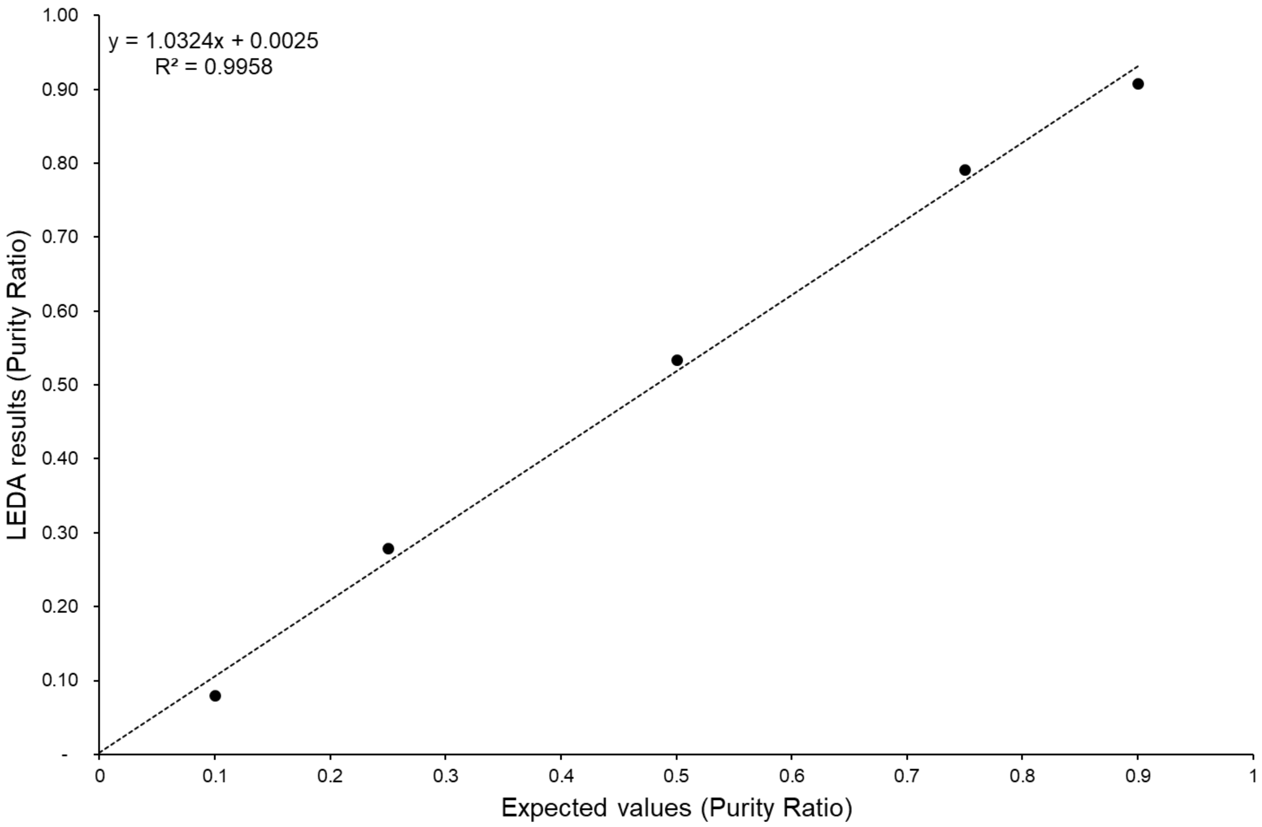

Figure S13. Validation plot obtained for the GDE5 isomer by processing the standard mixtures with $LEDA_{195\text{-}221}$ matrix.

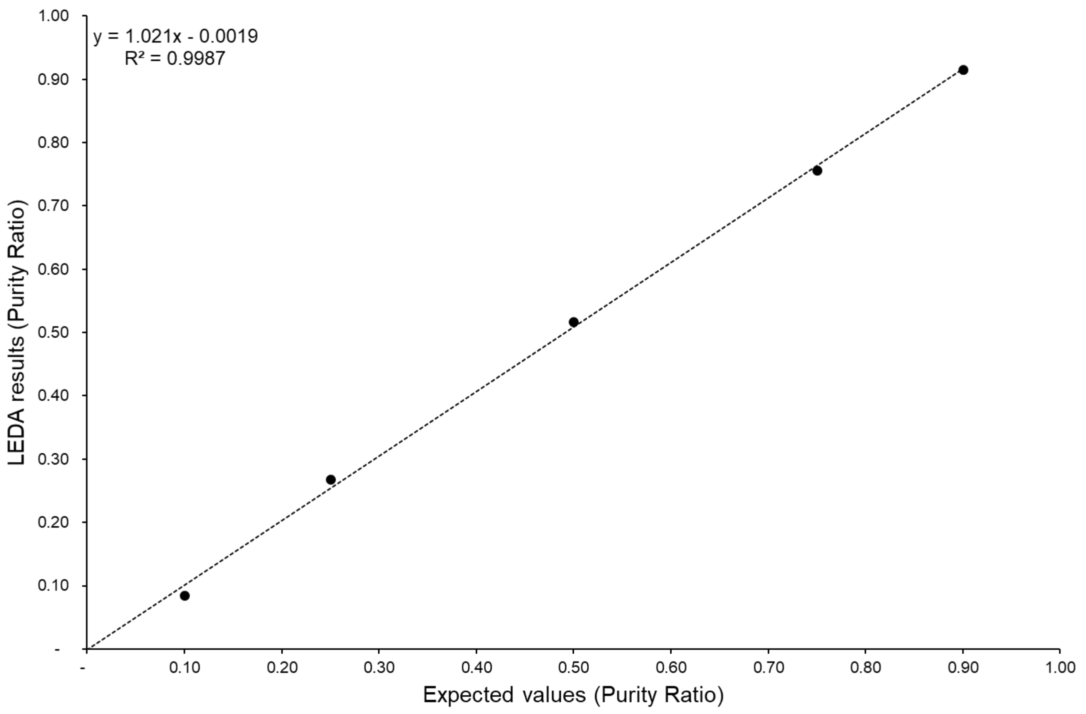

Figure S14. Validation plot obtained for the ELF94 isomer by processing the standard mixtures with $LEDA_{All}$ matrix.

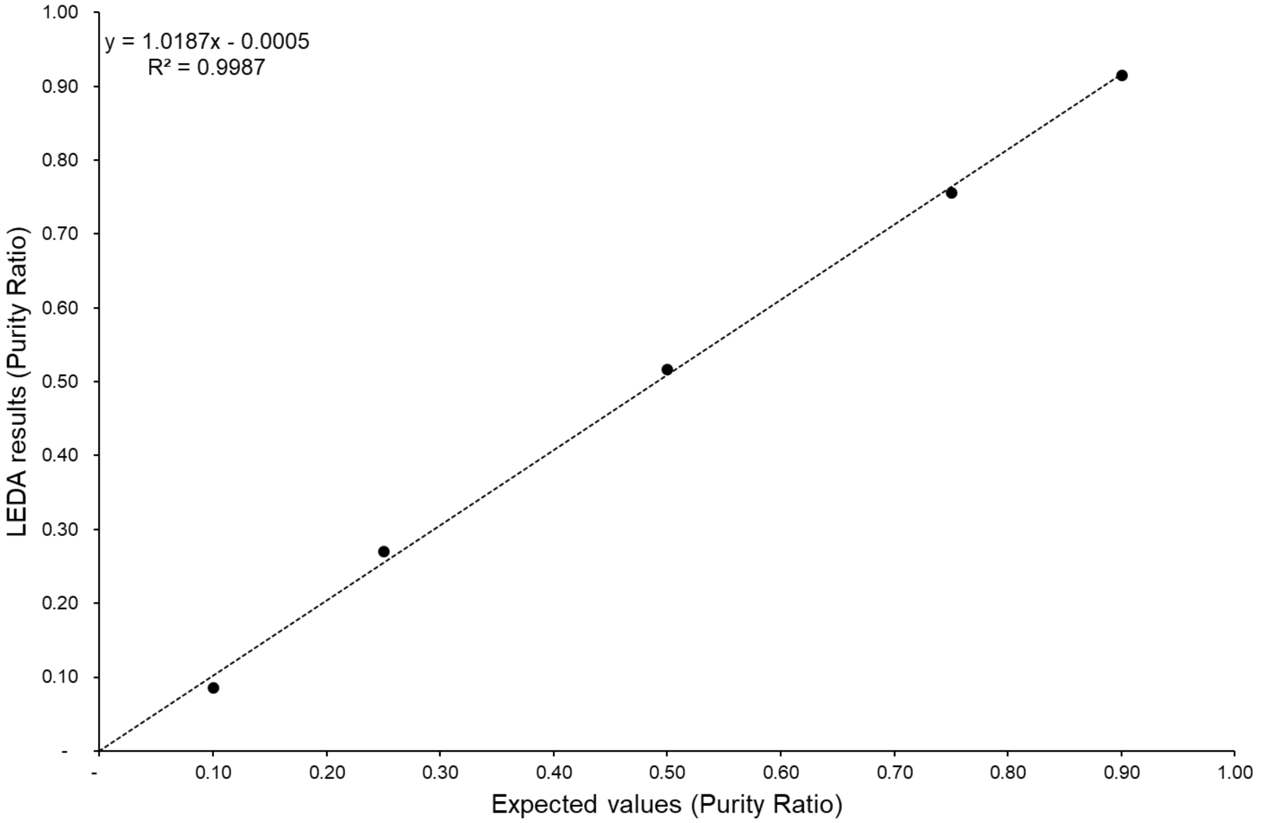

Figure S15. Validation plot obtained for the ELF94 isomer by processing the standard mixtures with $LEDA_{195\text{-}221}$ matrix.

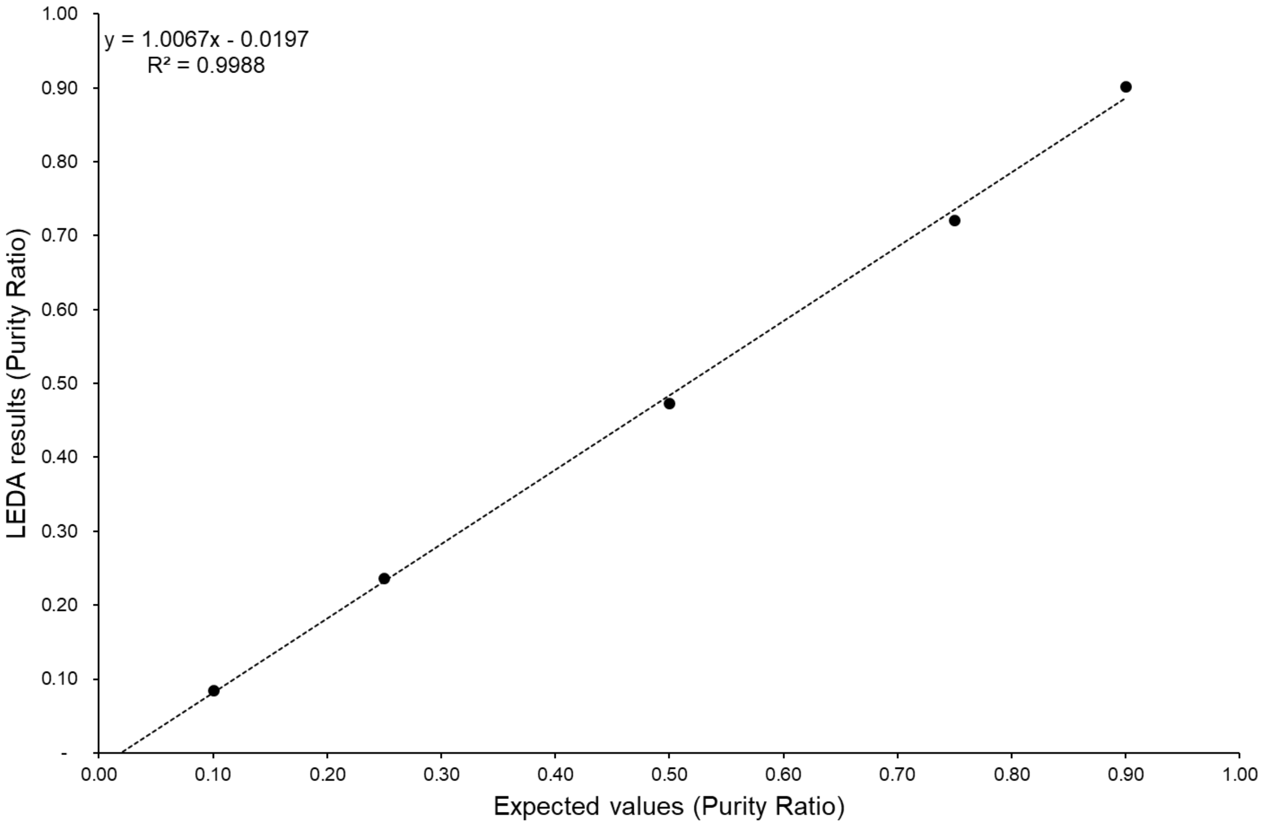

Figure S16. Validation plot obtained for the ELF96 isomer by processing the standard mixtures with $LEDA_{All}$ matrix.

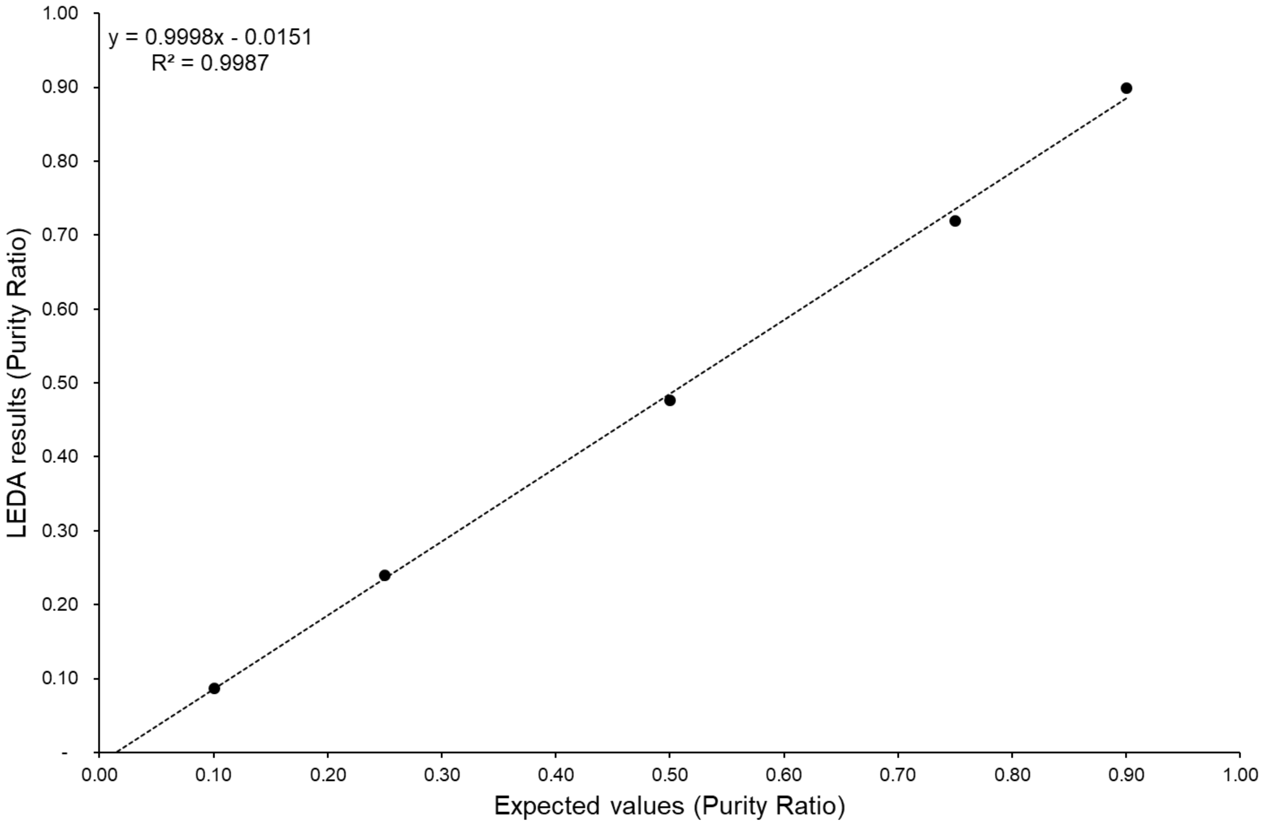

Figure S17. Validation plot obtained for the ELF96 isomer by processing the standard mixtures with $LEDA_{195\text{-}221}$ matrix.

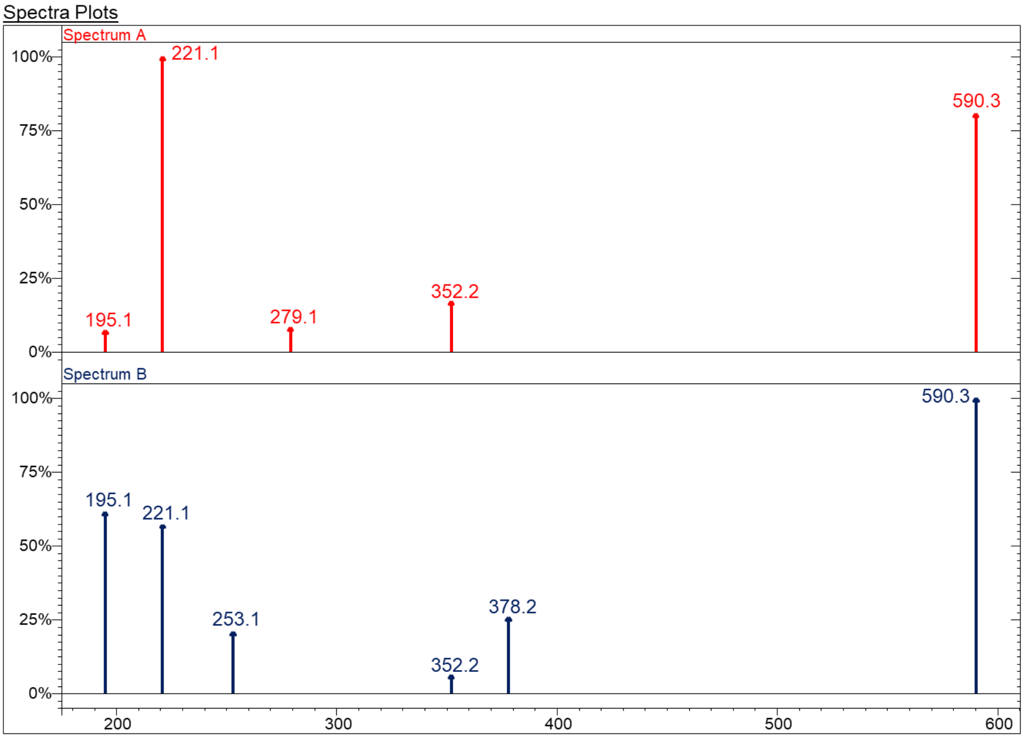

Figure S18. Extracted MRM spectra of the FRA76 (spectrum A) and GDE5 (spectrum B) isomers obtained by analyzing the 100.0 ng mL^{-1} calibration solution of each pure isomer with proper HPLC-MS/MS method.

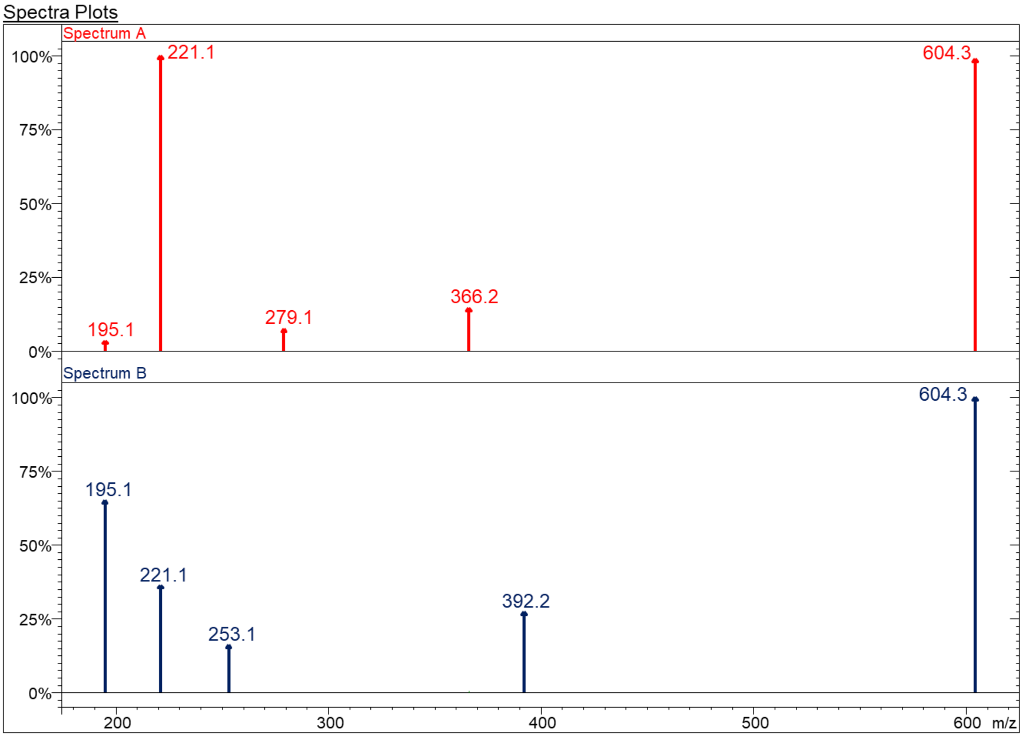

Figure S19. Extracted MRM spectra of the ELF94 (spectrum A) and ELF96 (spectrum B) isomers obtained by analyzing the 100.0 ng mL^{-1} calibration solution of each pure isomer with proper HPLC-MS/MS method.

Table S2. The results of calibration curves obtained for each MRM transition, defined as linear regressions parameters (slope and y-intercept), the determination coefficient (R^2) and the estimated LOD and LOQ values for each analyte.

Compound	MRM Transition	Slope (PAR/ng mL^{-1})	y-Intercept (PAR)	R^2	LOD (ng mL^{-1})	LOQ (ng mL^{-1})
FRA76	Ri	0.031	0.092	0.998	3.2	9.6
	Pi_1	0.040	0.114	0.998	3.3	9.9
GDE5	Ri	0.035	0.108	0.998	3.5	10.5
	Pi_2	0.022	0.072	0.999	3.4	10.2
ELF94	Ri	0.034	0.042	0.999	1.5	4.5
	Pi_1	0.038	0.043	0.999	1.8	5.4
ELF96	Ri	0.031	0.046	0.999	2.8	8.4
	Pi_2	0.020	0.029	0.999	3.3	9.9

Table S3. The calculated matrix effects (ME) and recovery (RE) values for IS and each analytes by using the proposed HPLC-MS/MS methods.

Compound	ME ± SD (%)	RE ± SD (%)
IS	101 ± 3	99 ± 2
FRA76	94 ± 8	99 ± 9
GDE5	100 ± 4	92 ± 2
ELF94	92 ± 3	87 ± 3
ELF96	88 ± 6	94 ± 8

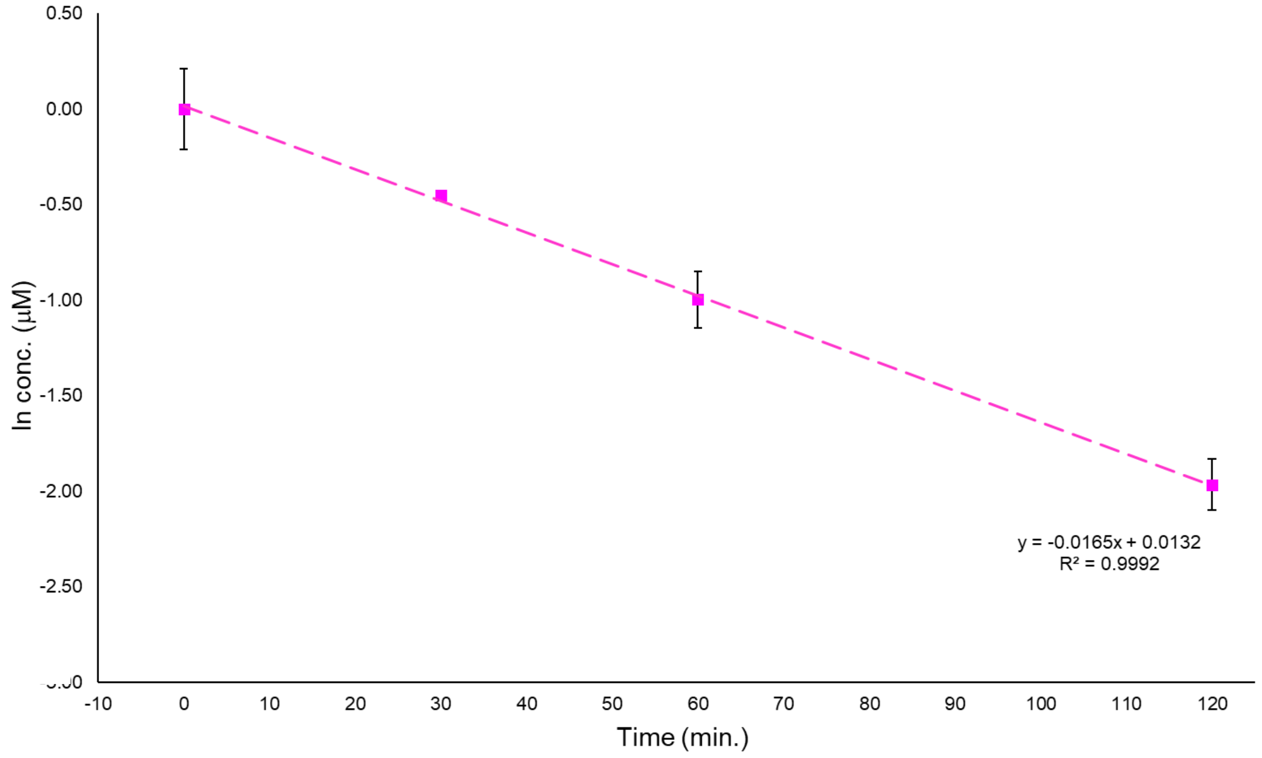

Figure S20. Degradation plots obtained by conventional quantitative method of the human plasma samples spiked with the FRA76 (pink square) isomer.

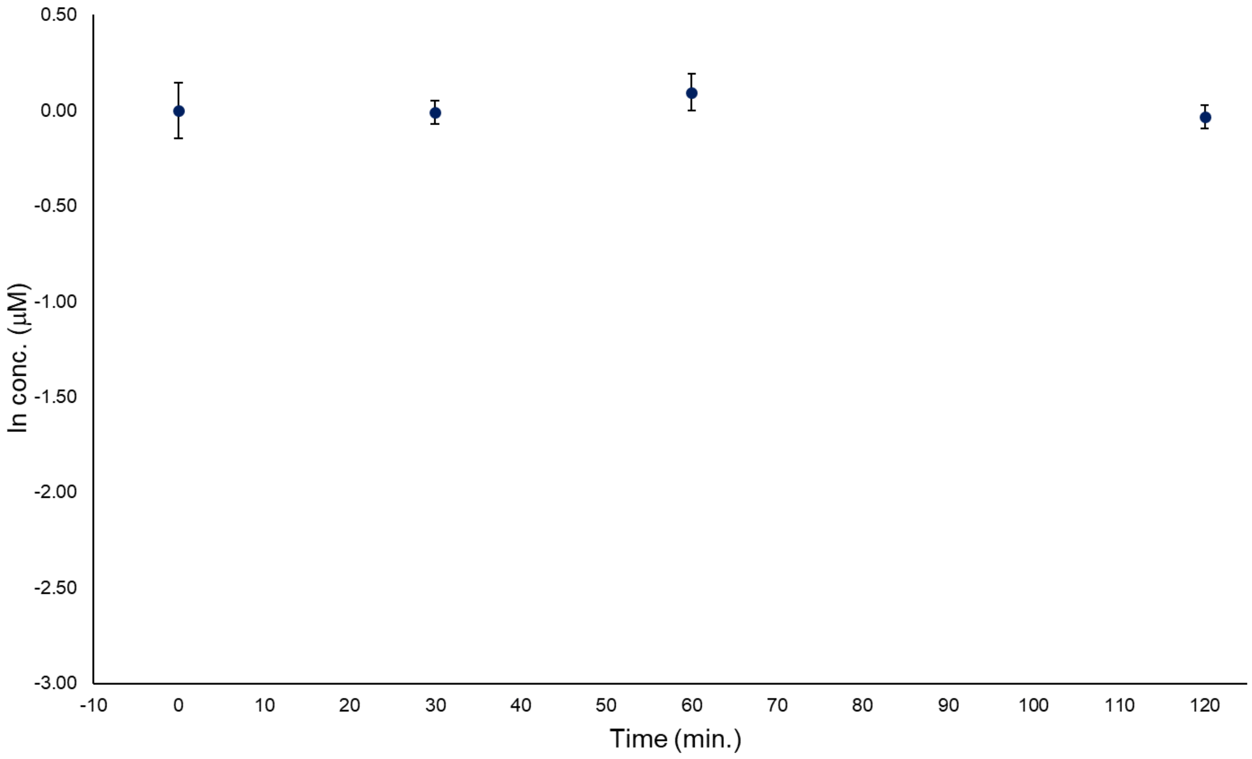

Figure S21. Degradation plots obtained by conventional quantitative method of the human plasma samples spiked with the GDE5 (blue circles) isomer.

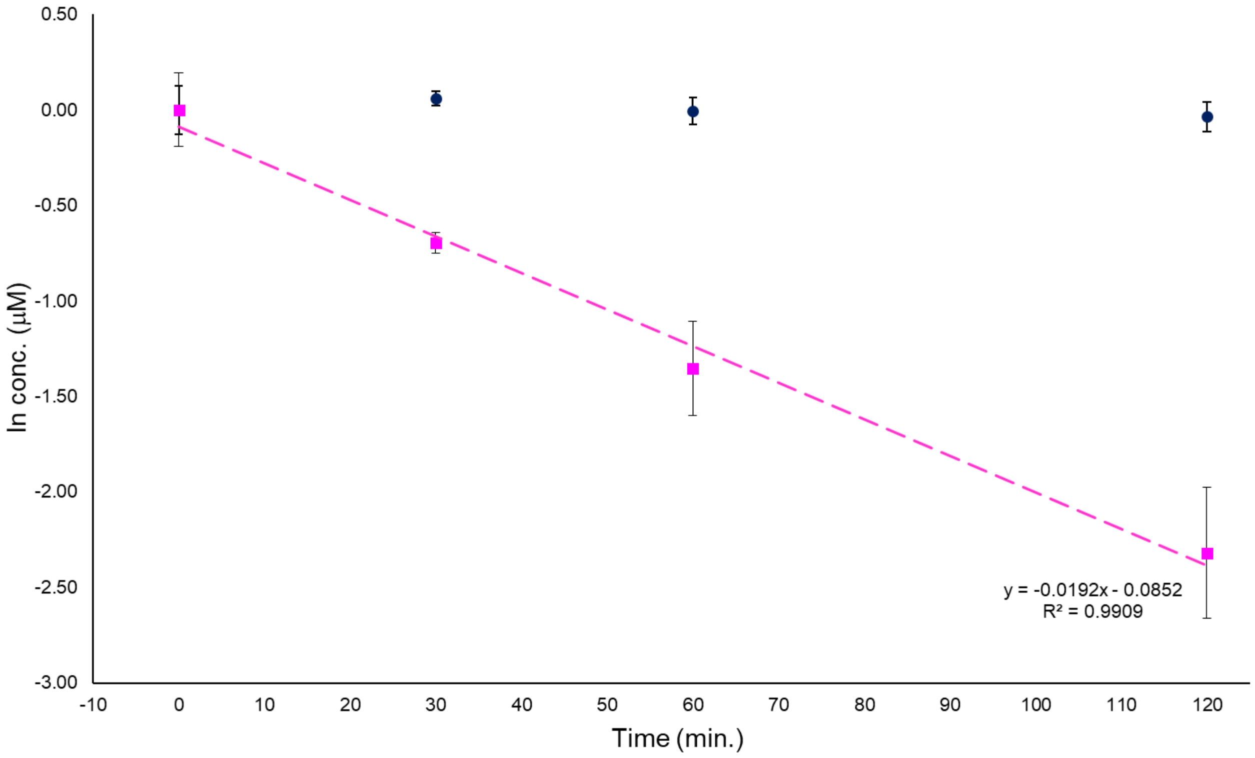

Figure S22. Degradation plots obtained by LEDA elaboration of the human plasma samples spiked with mixtures of the FRA76 (pink square) and GDE5 (blue circles) isomers.

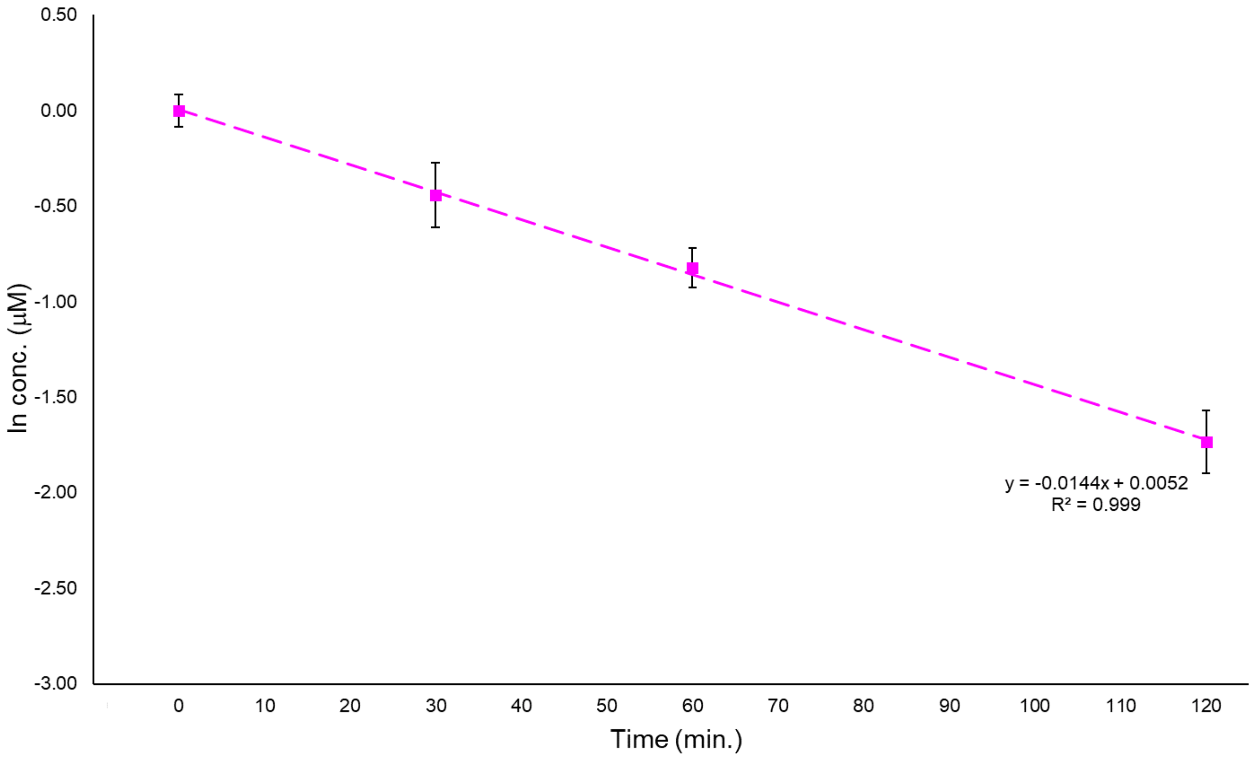

Figure S23. Degradation plots obtained by conventional quantitative method of the human plasma samples spiked with the ELF94 (pink square) isomer.

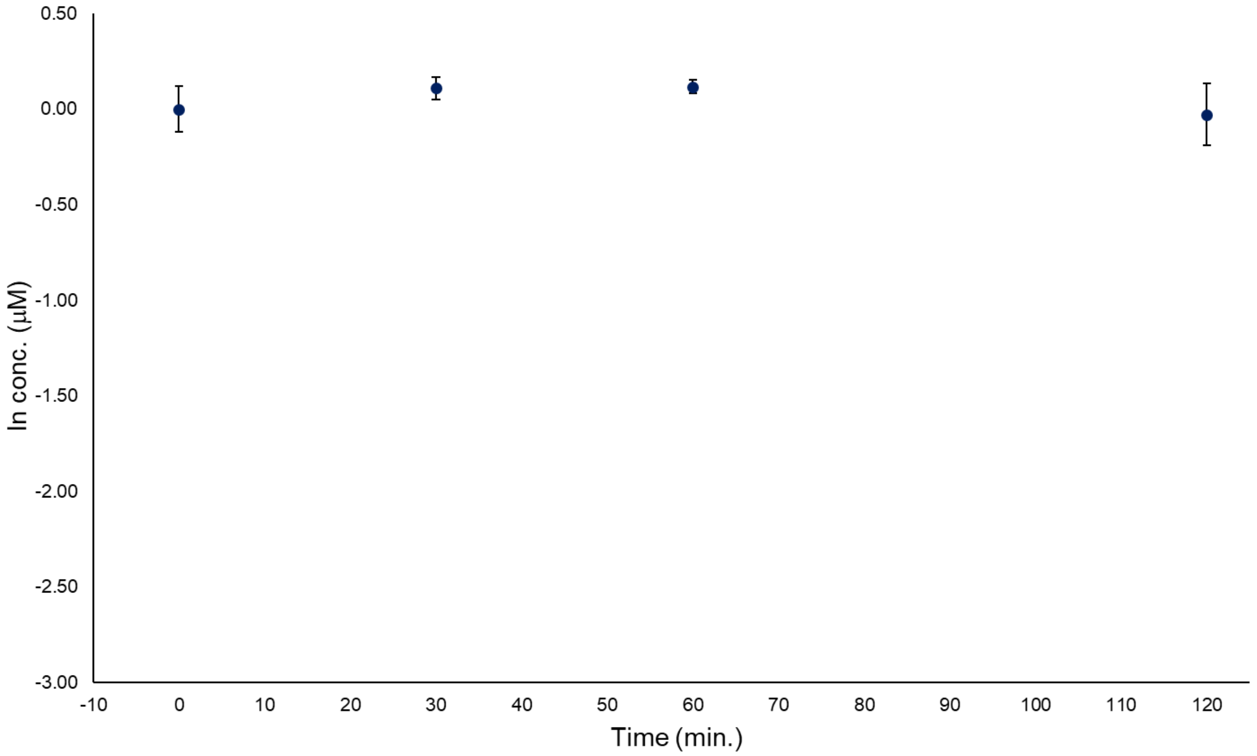

Figure S24. Degradation plots obtained by conventional quantitative method of the human plasma samples spiked with the ELF96 (blue circles) isomer.

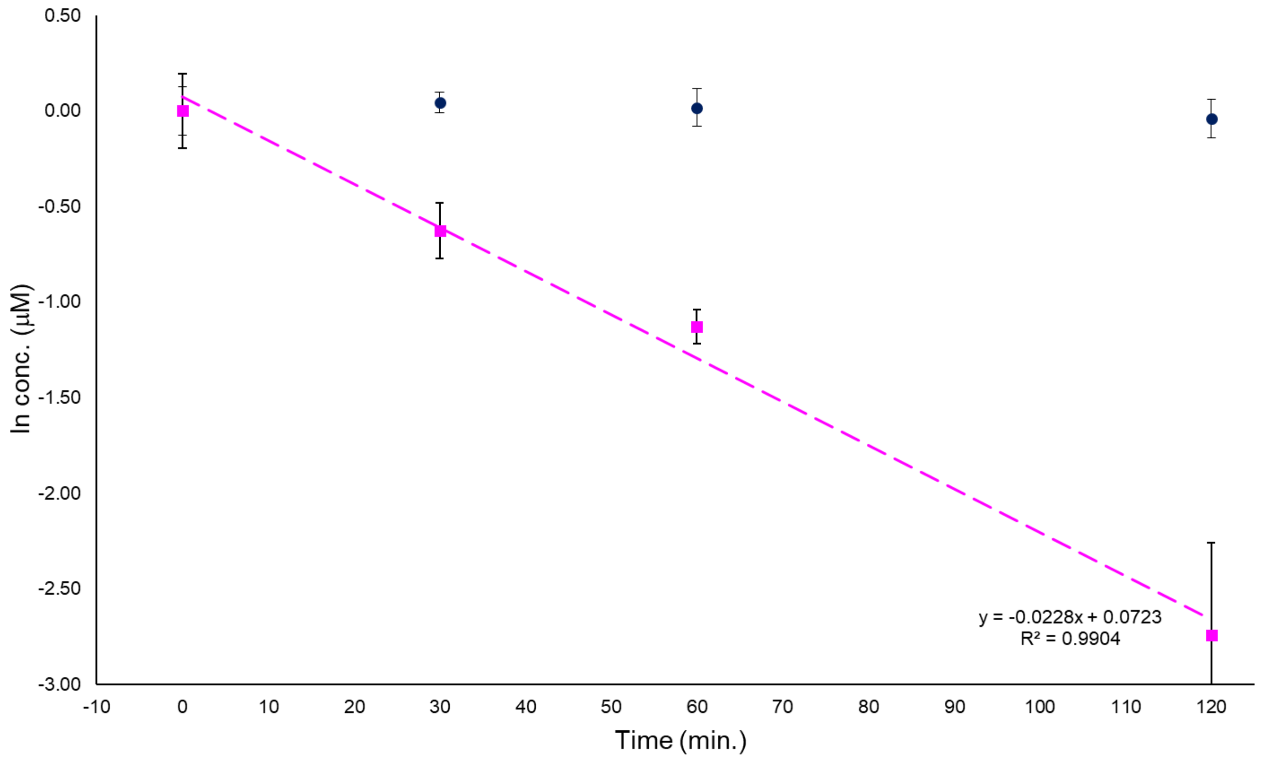

Figure S25. Degradation plots obtained by LEDA elaboration of the human plasma samples spiked with mixtures of the ELF94 (pink square) and ELF96 (blue circles) isomers.

Table S4. The characteristic degradation parameters, half time ($t_{1/2}$) and degradation rate (k), with the estimated standard deviations (SD), calculated by processing the MS/MS data with the proposed LEDA matrices from the series of human plasma samples spiked with isomers mixtures.

**Compound**	**LEDA$_{195–221}$ $t_{1/2}$ ± 2SD (min)**	**LEDA$_{19–221}$ k ± 2SD (ln(µM)/min)**	**LEDA$_{all}$ $t_{1/2}$ ± 2SD (min)**	**LEDA$_{all}$ k ± 2SD (ln(µM)/min)**
FRA76	32 ± 18	−0.019 ± 0.003	32 ± 19	−0.019 ± 0.005
GDE5	>120	<0.006	>120	<0.006
ELF94	34 ± 14	−0.023 ± 0.005	34 ± 15	−0.023 ± 0.003
ELF96	>120	<0.006	>120	<0.006

The LEDA reconstructed chromatographic profiles of all the sample involved in this study were reported below.

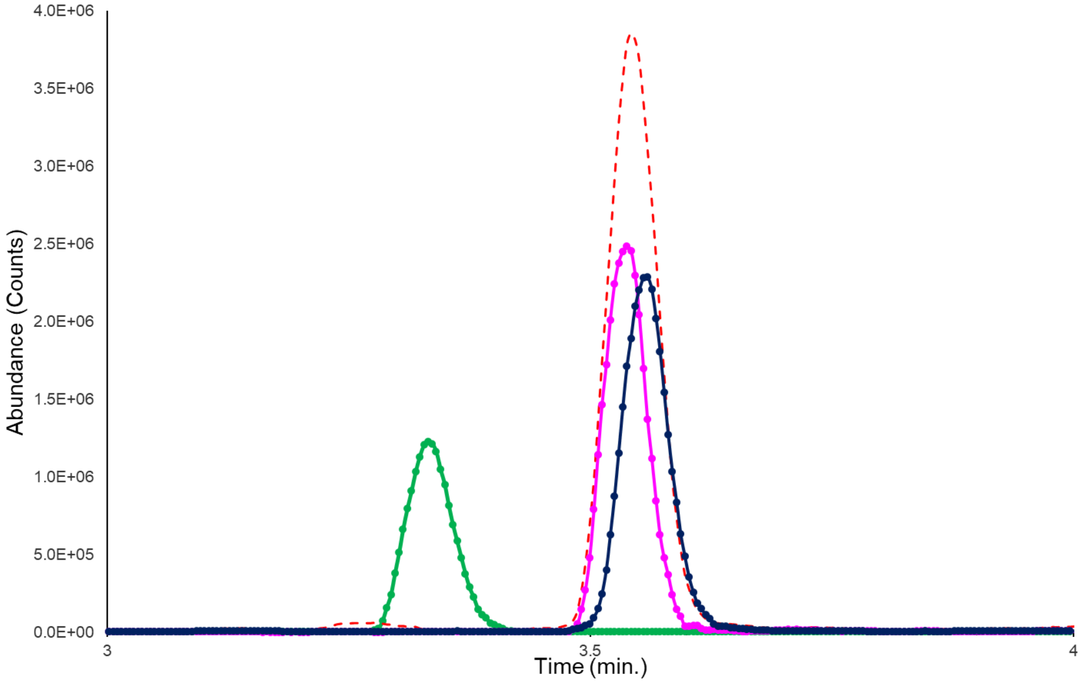

Figure S26. The LEDA reconstructed chromatographic profiles of the human plasma sample spiked with the mixture of the ELF94 (pink line) and ELF96 (blu line) isomers at incubation time 0 minutes. The IS (green line) and Ri (red dotted line) signals were also reported.

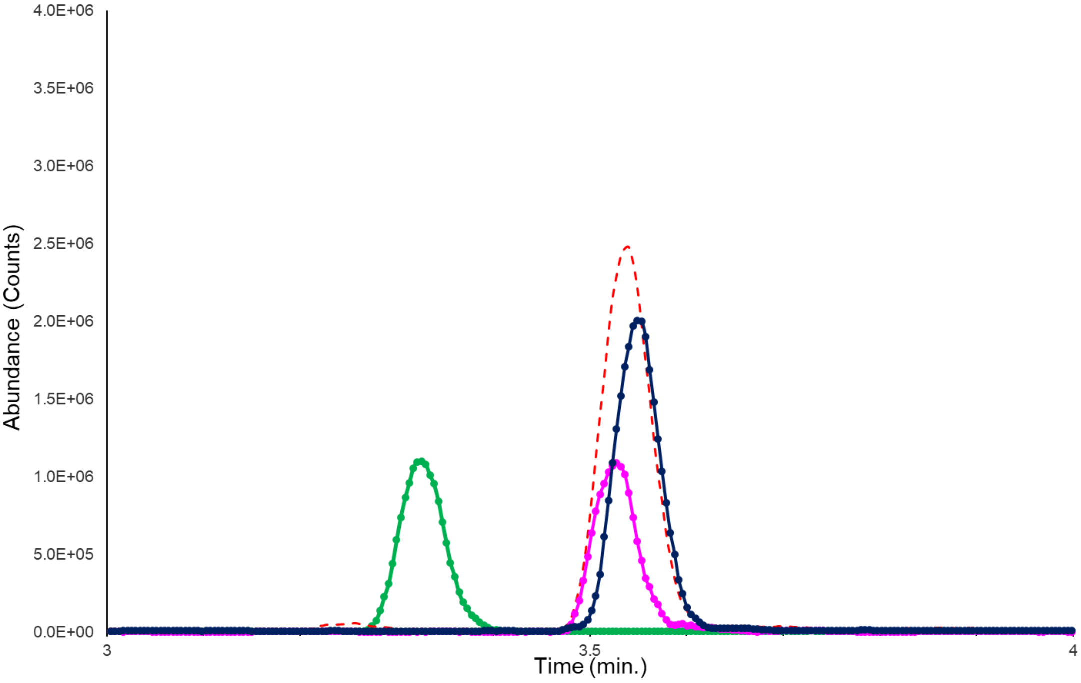

Figure S27. The LEDA reconstructed chromatographic profiles of the human plasma sample spiked with the mixture of the ELF94 (pink line) and ELF96 (blu line) isomers at incubation time 30 minutes. The IS (green line) and Ri (red dotted line) signals were also reported.

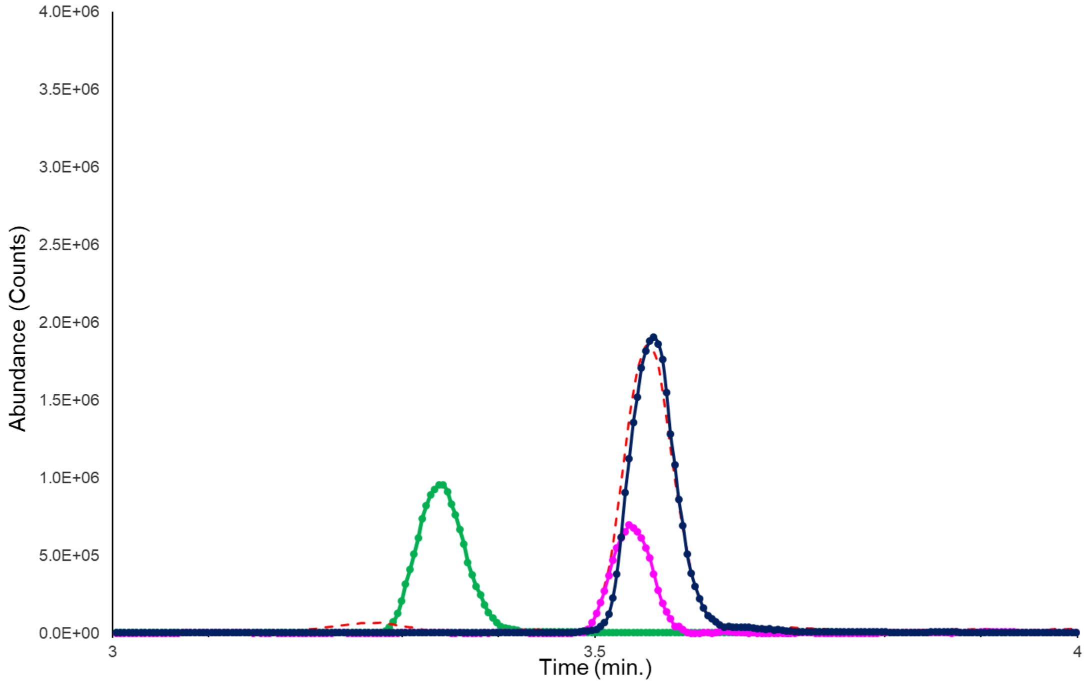

Figure S28. The LEDA reconstructed chromatographic profiles of the human plasma sample spiked with the mixture of the ELF94 (pink line) and ELF96 (blu line) isomers at incubation time 60 minutes. The IS (green line) and Ri (red dotted line) signals were also reported.

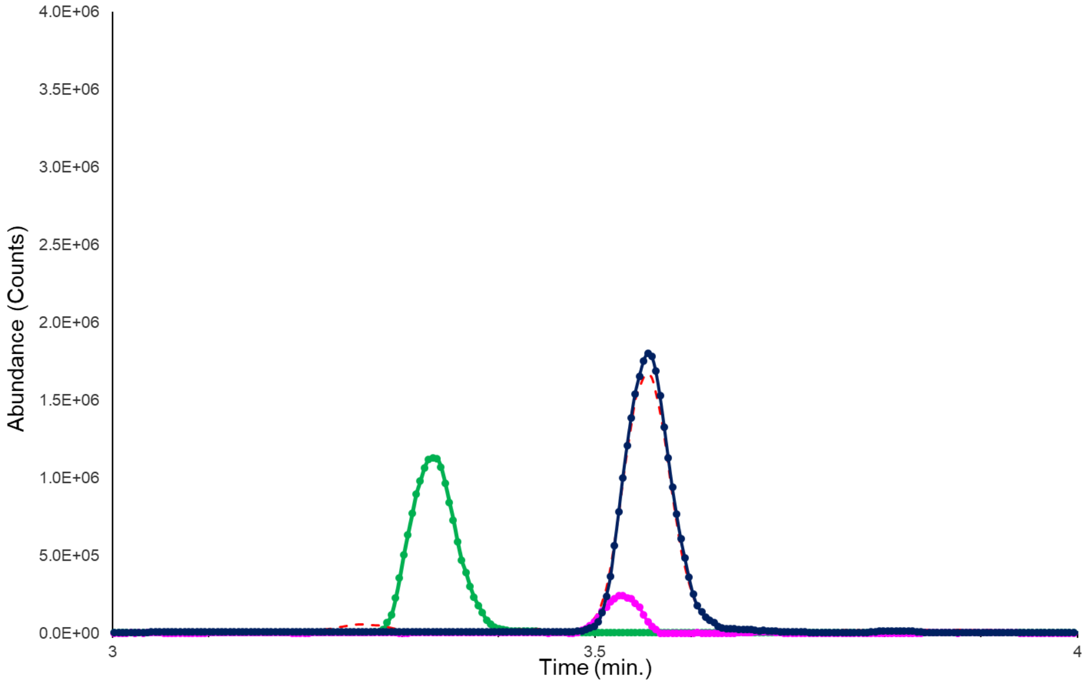

Figure S29. The LEDA reconstructed chromatographic profiles of the human plasma sample spiked with the mixture of the ELF94 (pink line) and ELF96 (blu line) isomers at incubation time 120 minutes. The IS (green line) and Ri (red dotted line) signals were also reported.

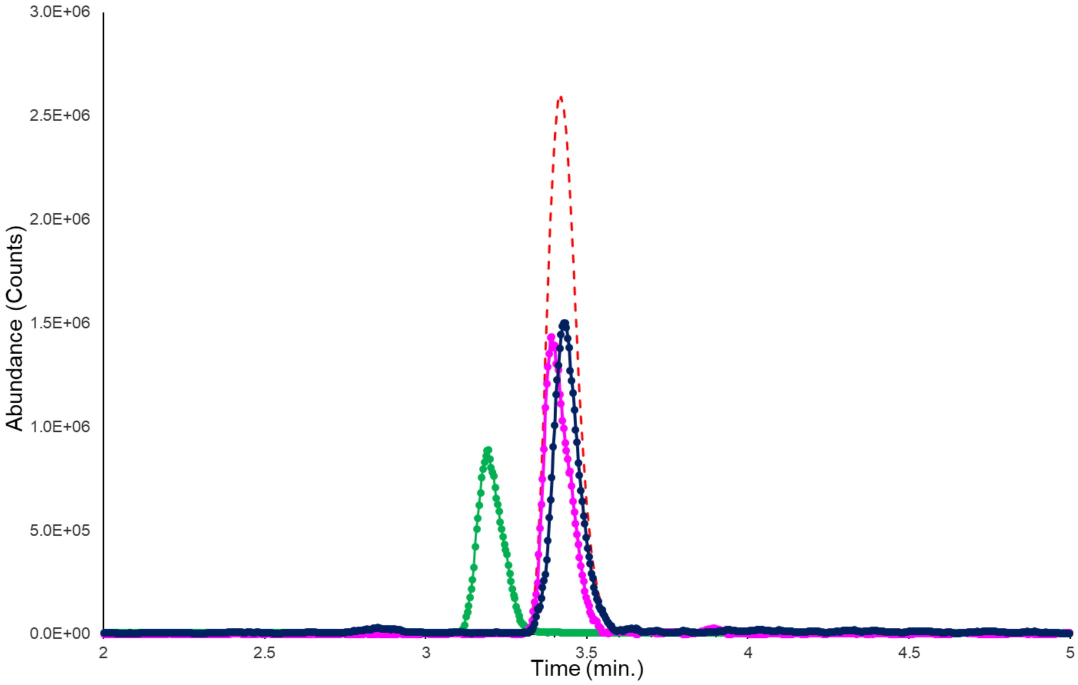

Figure S30. The LEDA reconstructed chromatographic profiles of the human plasma sample spiked with the mixture of the FRA76 (pink line) and GDE5 (blu line) isomers at incubation time 0 minutes. The IS (green line) and Ri (red dotted line) signals were also reported.

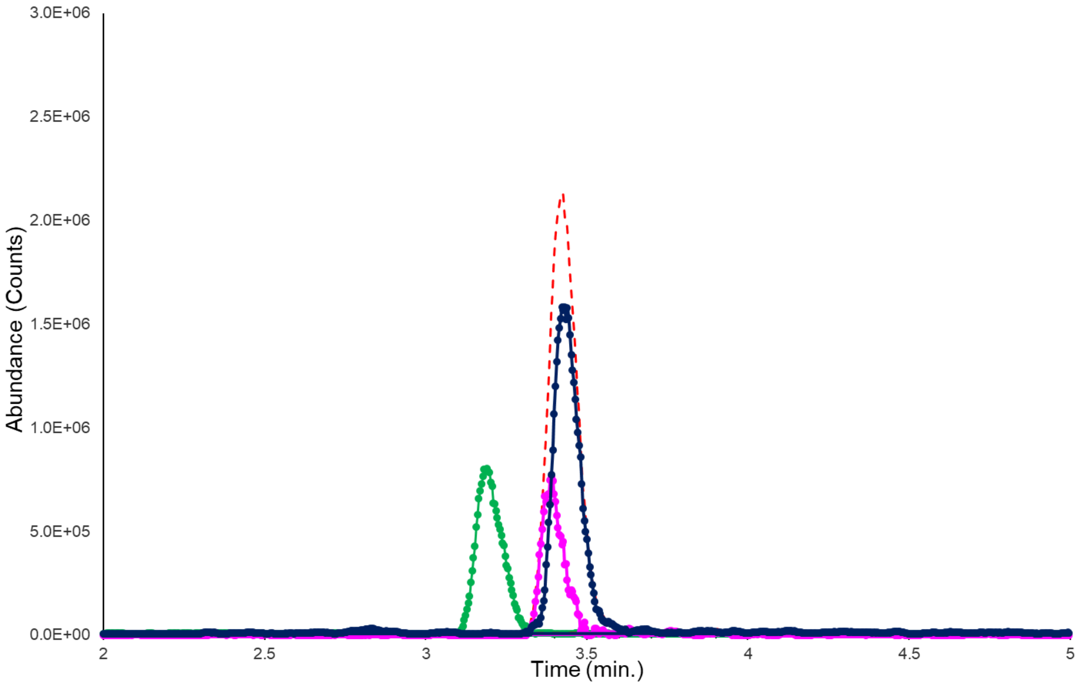

Figure S31. The LEDA reconstructed chromatographic profiles of the human plasma sample spiked with the mixture of the FRA76 (pink line) and GDE5 (blu line) isomers at incubation time 30 minutes. The IS (green line) and Ri (red dotted line) signals were also reported.

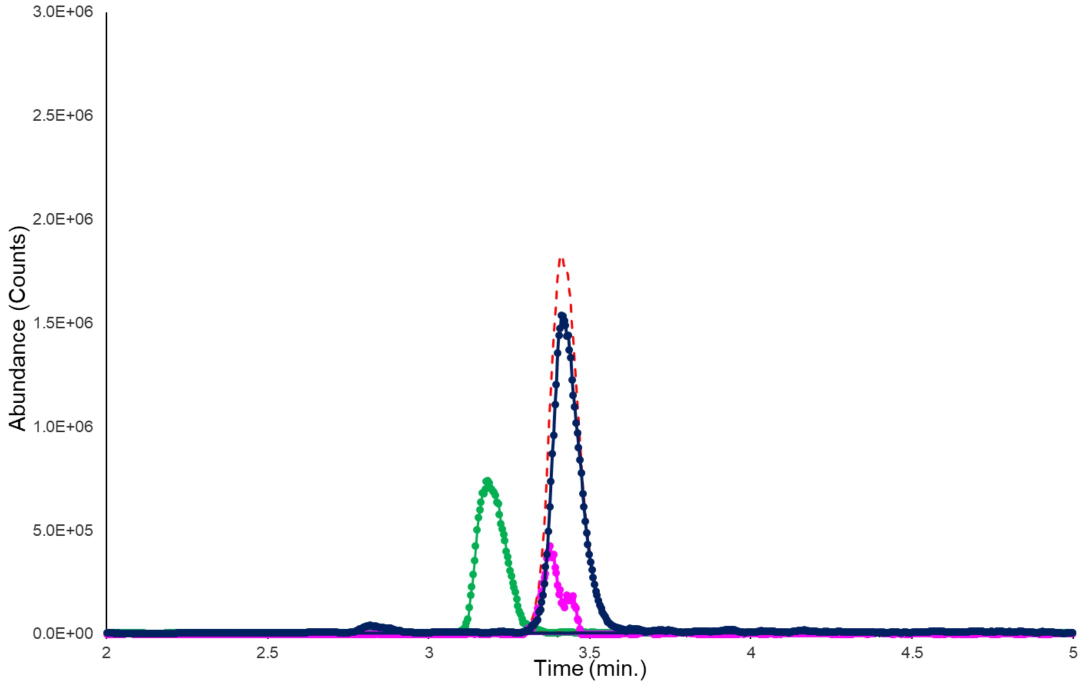

Figure S32. The LEDA reconstructed chromatographic profiles of the human plasma sample spiked with the mixture of the FRA76 (pink line) and GDE5 (blu line) isomers at incubation time 60 minutes. The IS (green line) and Ri (red dotted line) signals were also reported.

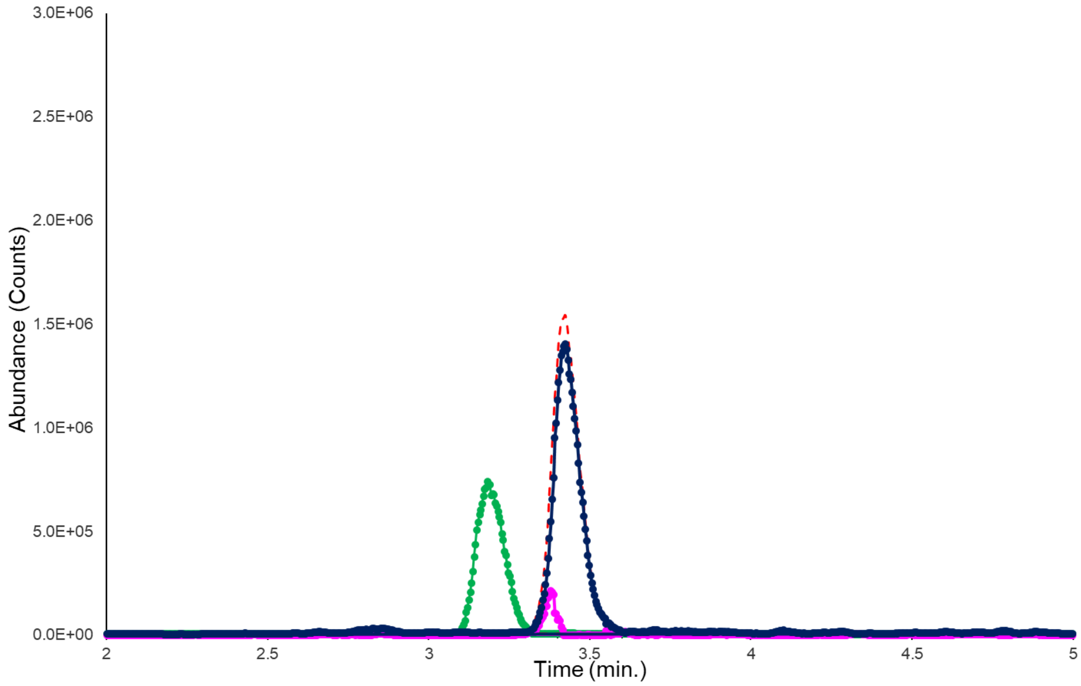

Figure S33. The LEDA reconstructed chromatographic profiles of the human plasma sample spiked with the mixture of the FRA76 (pink line) and GDE5 (blu line) isomers at incubation time 120 minutes. The IS (green line) and Ri (red dotted line) signals were also reported.

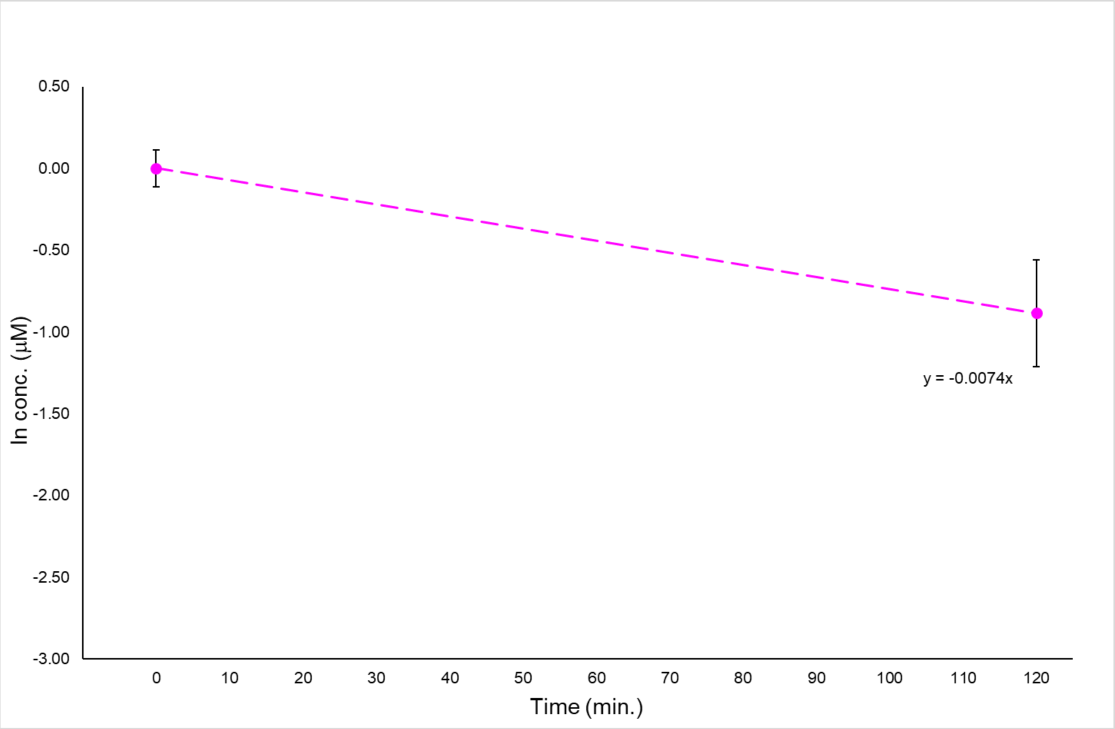

Figure S34. Degradation plots obtained by analysis with proper HPLC-MS/MS method of the human plasma samples spiked with the KEE reference compound.
